# Review: Degradable Magnesium Corrosion Control for Implant Applications

**DOI:** 10.3390/ma15186197

**Published:** 2022-09-06

**Authors:** Lifei Wang, Jianzhong He, Jiawen Yu, Srinivasan Arthanari, Huseung Lee, Hua Zhang, Liwei Lu, Guangsheng Huang, Bin Xing, Hongxia Wang, Kwang-Seon Shin

**Affiliations:** 1 Shanxi Key Laboratory of Advanced Magnesium-Based Materials, College of Materials Science and Engineering, Taiyuan University of Technology, Taiyuan 030024, China; 2 Chongqing Innovation Center of Industrial Big-Data Co. Ltd., National Engineering Laboratory for Industrial Big-data Application Technology, Chongqing 400707, China; 3 School of Materials Science and Engineering, Shanghai Jiao Tong University, Shanghai 200240, China; 4 Department of Mechanical & Materials Engineering Education, Chungnam National University, Daejeon 34134, Korea; 5 Institute for Advanced Studies in Precision Materials, Yantai University, Yantai 264005, China; 6 Hunan Provincial Key Laboratory of High Efficiency and Precision Machining of Difficult-to-Cut Material, Hunan University of Science and Technology, Xiangtan 411201, China; 7 National Engineering Research Center for Magnesium Alloys, College of Materials Science & Engineering, Chongqing University, Chongqing 400044, China; 8 Magnesium Technology Innovation Center, School of Materials Science and Engineering, Seoul National University, Seoul 881416, Korea

**Keywords:** biodegradable Mg alloy, corrosion resistance control, implants, mechanisms

## Abstract

Magnesium (Mg) alloys have received increasing interest in the past two decades as biomaterials due to their excellent biological compatibility. However, the corrosion resistance of Mg alloys is relativity low which limits their usage in degradable implant applications, and controlling the corrosion resistance is the key to solving this problem. This review discusses the relative corrosion mechanisms, including pitting, filiform, high temperature, stress corrosion, etc., of Mg alloys. Various approaches like purification (Fe, Ni, Cu, etc.), micro-alloying (adding Zn, Mn, Ca, RE elements, and so on), grain refinement (severe plastic deformation, SPD, etc.), and surface modifications (various coating methods) to control corrosion and biological performance are summarized. Moreover, the in vivo implantations of Mg alloy vascular stents and the issues that have emerged based on the reports in recent years are introduced. It is recommended that corrosion mechanisms should be further investigated as there is no method that can remove all the impurities and a new purification approach needs to be developed. The concentration of micro-alloy elements should be carefully controlled to avoid superfluous compounds. Developing new continuous SPD methods to achieve fine-grained Mg alloys with a large size scale is necessary. The development of a multifunctional coating could also be considered in controlling the Mg degradation rate. Moreover, the research trends and challenges in the future of Mg biomaterials are proposed.

## 1. Introduction

Due to the high specific strength, good machinability, and similar young’s modulus with the natural bone of the human body [1,2], Mg and its alloy can be used as an ideal bone plate material as well as a vascular stent. Besides, Mg is also a necessary trace element, participating in the whole process of metabolism, which plays a significant role in sustaining the biological activity of the human enzyme system [3,4]. For example, during the implantation as a stent, Mg can be degraded gradually so that the occurrence of vascular restenosis and the second removal operation are avoided. The surgical risk and economic burden for patients are reduced as well [5,6]. 

However, at present, there are still many shortcomings in Mg biomedical materials, limiting their wide applications. As is well known, the standard electrode potential of Mg and its alloys (−2.37 V) is low, which results in poor corrosion resistance and makes it easy to be corroded in the human environment, especially in the presence of a large number of aggressive Cl^−^ ions in biological fluids [7]. A higher corrosion rate will result in the premature failure of Mg alloy biomedical devices before reaching the service life and loss of mechanical stability, which is unable to support the diseased blood vessels and bones. Moreover, a large amount of hydrogen will be produced in the human body due to the rapid degradation rate. If the hydrogen cannot be released in time, a series of clinical problems will emerge, such as emphysema and local alkalinity of the internal environment. Therefore, obtaining biomedical Mg alloys with good biodegradability to meet the service requirements has become an urgent problem in recent years. In this review, various technologies to enhance the corrosion property of biological Mg and its alloys are summarized, and the insufficiencies are analyzed. Finally, the research line in the future is suggested.

## 2. Corrosion Mechanisms of Mg and Its Alloys

In general, there are three major factors are responsible for the low corrosion resistance of Mg and its alloy: (1) Mg has a negative reduction potential so that the electrochemical reactions in an aqueous solution are undergoing spontaneously, (2) the electrode potential of Mg and the current density of hydrogen evolution exchange is relatively low, the corrosion current density of the alloy may increase significantly when a corrosive microcell with other metals is formed, and (3) due to the loosely bound corrosion products and the release of H_2_ during the reaction, the oxidation film and the products formed during corrosion are unable to provide protection to the matrix alloy, leading to the rapid rate of corrosion on Mg [8,9]. 

**Figure 1:**
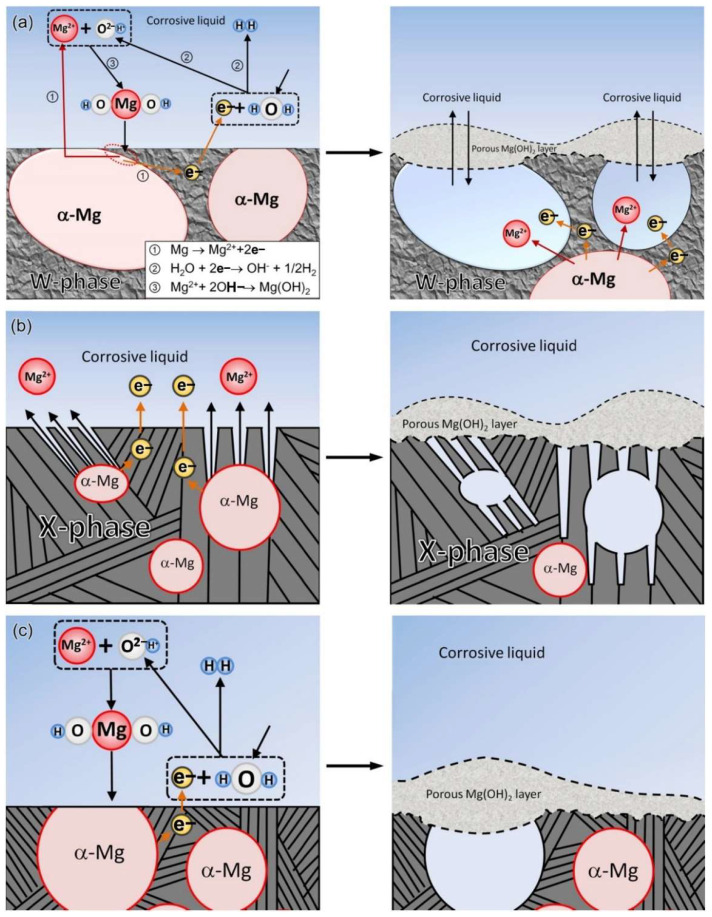
Schematic diagram of the corrosion mechanism: (**a**) MgY1.72Zn2.81Zr0.17 alloy, (**b**) MgY3.83Zn3.03Zr0.17 alloy, (**c**) MgY2.58Zn1.27Zr0.15 alloy [10].

Common corrosion types for Mg alloy includes galvanic corrosion, pitting corrosion, filamentous corrosion, etc., which are summarized in this part. Figure 1 illustrates the corrosion mechanism of Mg alloys [10]. The electrochemical reaction that takes place during Mg corrosion is as follows:

Response of anode: Mg → Mg^2+^ + 2e^−^ (1)

Response of cathode: 2H_2_O + 2e^−^ → H_2_ ↑+ 2OH^−^ (2)

Or: 2H^+^ + 2e^−^ → H_2_ ↑ (3)

Net reaction: Mg + 2H_2_O → Mg(OH)_2_ + H_2_↑ (4)

### 2.1. Galvanic Corrosion 

Galvanic corrosion usually occurs in Mg alloys. In most of the Mg alloys during corrosion, the Mg matrix act as an anodic phase, while the second phases and impurities in the alloy are cathodes [11]. The main reason for Mg corrosion in an aqueous solution is that H^+^ or H_2_O molecules act as oxidants. The equilibrium potential of their reduction reaction is higher than that of the oxidation reaction of Mg. Therefore, the differences in free energy for the electrochemical reactions are negative. The reaction of the Mg electrode in an aqueous solution can be spontaneous. Some regions of the Mg matrix undergo an oxidation reaction from Mg atoms to Mg^2+^ due to its anodic characteristics. These released ions transfer to the cathode, combine with H^+^ or H_2_O molecules in the solution, and undergo a cathodic reaction [12]. The degree of galvanic corrosion on Mg alloys is mainly related to the solution composition of the corrosive medium, alloy composition, and environmental conditions. 

### 2.2. Local Corrosion

(1) Pitting Corrosion

Passive metals exhibit excellent general corrosion properties; however, it is much easier to be corroded by pitting attack in a certain aggressive solution, which results in the failure of materials [13]. Mg alloy is a naturally passivated metal. When Cl^−^ is encountered in a non-oxidizing medium, it tends to be corroded and cracked, and corrosion occurs outside its free corrosion potential. The main reason for the pitting corrosion is the formation of blocked cells and the acidification autocatalysis in the hole [14]. During the initial stage of pitting, the formation of cells with local oxygen concentration differences results in the surrounding solution being more acidic. Further, as the concentration of cations increases, the solution will not remain electrically neutral. Thus, the negatively charged Cl^−^ enters the etched hole and combines with the cation in the membrane to form a soluble chloride. Finally, a certain number of small corrosion holes are formed at a certain point on the metal matrix [15]. In the growth stage, the metal surface in the pitting is more negative and acts as an anode. On the other hand, the metal surface on the pitting corrosion is blunt, and the potential is positive, which acts as a cathode. Therefore, an activated and passivated micro galvanic corrosion battery is formed inside and outside the hole, and the metal outside the pitting is protected. Pitting corrosion of Mg alloys occurs in neutral or alkaline solutions, and heavy metal pollution also accelerates the pitting corrosion [16].

(2) Filiform Corrosion

**Figure 2:**
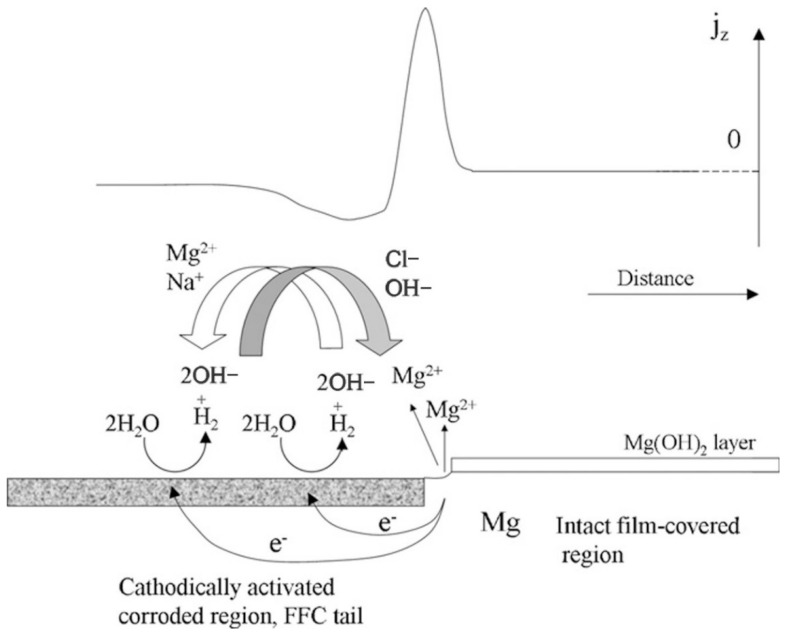
Schematic representation of FCC mechanism on magnesium under immersion conditions [18].

The filiform corrosion mainly happens due to the active etching cell moving towards the grain boundary surface, with the anode at the head and the cathode at the tail. Filiform corrosion usually emerges on the protective coating as well as the anodized layer, and the Mg without coating does not suffer from filiform corrosion much [17]. In the case of AZ91 Mg alloys, pitting begins at certain points on the surface layer with many intermetallic compounds. The filamentous corrosion is controlled by a strong anode on the surface. The corrosion degrees are related to temperature, material structure characteristics, surface treatment, etc. The process of filiform corrosion is represented schematically in Figure 2 [18]. 

### 2.3. High-Temperature Corrosion

Mg alloy can easily be oxidized in air, and MgO is not protective at high temperatures. Besides, the corrosion rate increases much faster with increasing temperature than that of pure Mg [19]. This is mainly due to the high temperature, which enhances the activity of even a small amount of impurity in the alloy. However, the Mg-RE system has a better high-temperature corrosion resistance [20]. 

### 2.4. Stress Corrosion

**Figure 3:**
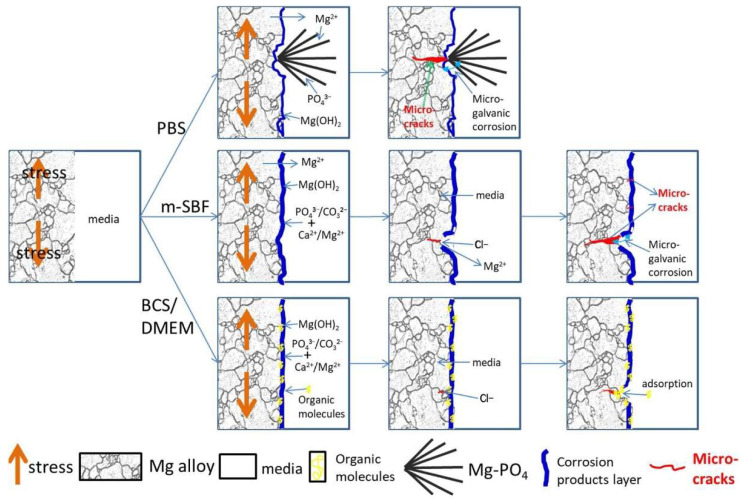
Schematic diagrams of the stress corrosion cracking (SCC) behavior of Mg-1Zn alloy in PBS, m-SBF, DMEM, and BCS media [23].

Stress corrosion cracking (SCC) is related to the fracture of metals under the combined tensile stress and an environment which are highly sensitive to Mg alloys. Stress corrosion cracking often occurs after stress corrosion for a period [21]. Most of the solutions chosen for the theoretical study of corrosion cracking are chloride and chromate. Previous reports indicated that the degradation environment had an important effect on the in vitro SCC behavior of Mg-1Zn alloy [22]. Figure 3 illustrates the schematic diagrams of the stress corrosion cracking behavior of Mg-1Zn alloy in PBS, SBF, DMEM, and BCS media [23]. In PBS, the micro-galvanic corrosion formed by the outward growth flocculent product accelerates the formation of stress concentration sites and hydrogen; in SBF, the pitting corrosion and break of the surface film under deformation lead to a high SCC susceptibility; in DMEM and BCS, the homogeneous corrosion behavior and adsorption of organic component into micro-tunnel retard the formation of stress initiation sites and electrolyte exchange then decreasing the propagation of SCC.

### 2.5. Corrosion Fatigue

Corrosion fatigue is a brittle fracture of metal under cyclic stress in a corrosive medium. For Mg alloy, it is more likely to occur in the corrosive medium than in the air. Previous studies have shown that the fatigue life of casting and extruded AZ91D, AZ31, and AM50 Mg alloys are significantly reduced in a 3.5% NaCl solution [24]. The difference in corrosion fatigue properties in various environments can be explained by their mechanical and chemical behaviors. Zeng et al. [25] studied the corrosion fatigue of modified AM80 in sodium chloride solution. The results indicated that the corrosion properties varied in different solutions, and Cl^−^ was one of the main reasons for the decrease in corrosion fatigue life. 

## 3. Approaches to Magnesium Corrosion Control

Mg alloys can corrode through several mechanisms, as discussed in Section 2. Several factors such as purity, alloy elements, grain size, protective film, and so on will also influence the corrosion properties of Mg and their mechanism. How these factors affect corrosion behaviors and the relative corrosion controlling methods are important. This part summarizes various approaches to control the corrosion behaviors of Mg alloys in detail. 

### 3.1. Purification of Mg Alloys 

The impurity elements, such as Fe, Ni, Cu, etc., present in Mg alloys generally accelerate their corrosion. The solid solubility of several elements in Mg alloys is very low, and they usually form intermetallic compounds. When the concentration is less than 0.2%, it will have a harmful effect on the corrosion rate of Mg alloys. Dai et al. [26] showed that the increase in corrosion rate caused by Fe was mainly due to the formation of an electric couple between Fe and Mg. While the solid solubility of Ni in Mg alloys was very low when added as an independent phase [27]. A small amount of Cu and Mg can form intermetallic compounds, which distribute around the grain boundary and accelerates the corrosion rate effectively [28]. 

To enhance the corrosion resistance of Mg alloy, the content of impurity elements in raw materials and smelting should be controlled strictly. Gain et al. [29] suggested the allowable limit of impurity elements for Mg alloy in NaCl solution: Fe ≤ 0.032 × Mn%, Ni ≤ 0.001%, and Cu ≤ 0.04%. The corrosion resistance may not be significantly affected in this range of impurities. Zhang et al. [30] studied the corrosion behavior of pure Mg and Mg-6Mn alloy in 0.6 M NaCl solution. They found that the cathodic activation behavior of pure Mg was suppressive in Mg-Mn alloy induced by Mn(Fe) phase. Then the corrosion resistance of Mg-Mn alloy was enhanced. Wang et al. [31] indicated that the corrosion resistance of AZ91 Mg alloy was better when the Fe/Mn ratio was less than 0.032. Göken et al. [32] compared the salt spray corrosion properties of high purity and common AZ91 alloy. They found that the corrosion rate of common AZ91 alloy was 19 times that of high purity AZ91 alloy. Liu et al. [33] explored the effect of casting temperature on the content of impurity Fe in Mg alloy. It was found that the content of Fe can be reduced to a great extent when the casting temperature was between 630–680 °C. With the decrease in casting temperatures, the insoluble second phase in the alloy decreased, which resulted in the improvement of corrosion resistance. Therefore, the selection of high purity raw materials, improving the melting process, and reducing the content of impurities during the melting process are possible methods to enhance the corrosion resistance of biological Mg alloys. 

**Figure 4:**
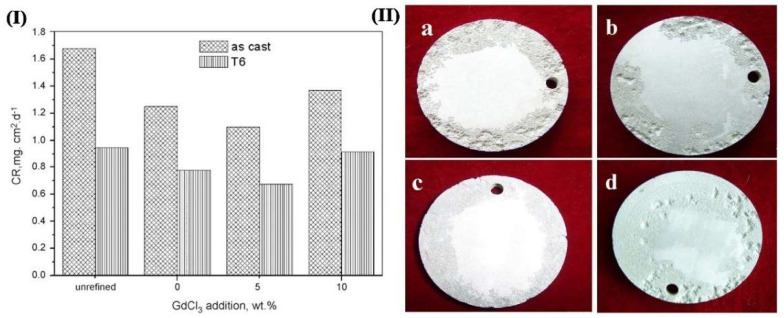
(**I**) corrosion rates of GW103K alloy; (**II**) Surface corrosion morphologies after immersed in 5 wt.% NaCl for 3 days ((**a**) unrefined, (**b**) refined by JDMJ, (**c**) refined by JDMJ + 5 wt.% GdCl3 additions, (**d**) refined by JDMJ + 10 wt.% GdCl3 additions) [35].

What is more, to purify the Mg alloy, the best method is metal thermal reduction. Some halides of highly active metals such as Ti, Zr, and Mn are added to the alloy to react with Mg, forming insoluble and refractory metal compounds, and these metal compounds can be precipitated and reduce the impurities. For example, Wu et al. [34] found that Mn can form the MnFe phase with impurity Fe in the alloy, which precipitated at the bottom of the crucible before casting, reducing the content of impurity Fe. Wang et al. [35] found that the corrosion rate of GW103K alloy decreased to 0.437 mg/cm^−2^ d^−1^ after JDMJ + 5 wt.% GdCl_3_ refinement through 5 wt.% NaCl immersion experiment. In Figure 4, the corrosion rate reduced as the order: no refining, pure JDMJ, JDMJ − 10 wt.% GdCl_3_, JDMJ −5 wt.% GdCl_3_ samples. The morphological characteristics of the specimen surface after three days of immersion in 5 wt.% NaCl solution is shown in Figure 4. There were many deep corrosion pits emerging on the surface. However, it was fine on the sample refined with 5 wt.% GdCl_3_. The degree of micro-galvanic corrosion was reduced due to the decrease of impurity elements, and the corrosion resistance was improved. However, there are many kinds of impurities in Mg alloys, including element and compound inclusions. Although many purification methods for Mg and Mg alloys have been applied, there is no method that can remove all the impurities. The approach needs to be further investigated deeply. 

### 3.2. Micro-Alloying

Micro-alloying is a traditional approach to enhancing the corrosion resistance of Mg alloys. By adding micro-elements to the Mg alloy, the second phase at the grain boundary can be prevented effectively, and the electrode potential of the alloy will be increased. The potential differences between the Mg matrix and the second phase decrease. This reduces the cathodic reaction rate and promotes the uniform distribution of galvanic corrosion current on the anode surface. Thus the local corrosion is inhibited [36]. The addition of alloying elements with high hydrogen evolution overpotential can increase the cathodic potential of the alloy, reducing the corrosion properties of the alloy in acid solution. Meanwhile, micro-alloying can also reduce the anode activity of Mg alloys. For example, adding easily passivating elements to the alloy can improve the self-passivating ability of the alloy [37]. The alloy forms a complete and compact passivating film on the surface so that its corrosion rate will decrease. In addition, the concentration of alloying elements increases the density of oxide film, which has a strong protective effect and enhances corrosion resistance. Al, Mn, Zn, Cr, Sr, and RE are considered ideal alloying elements. Besides, the corrosion resistance of biological Mg alloys usually increases first and then tends to decrease with the increasing number of alloying elements. The effect of some common alloying elements on the corrosion properties of biological Mg alloy is introduced briefly in the following part.

#### 3.2.1. Zinc

**Figure 5:**
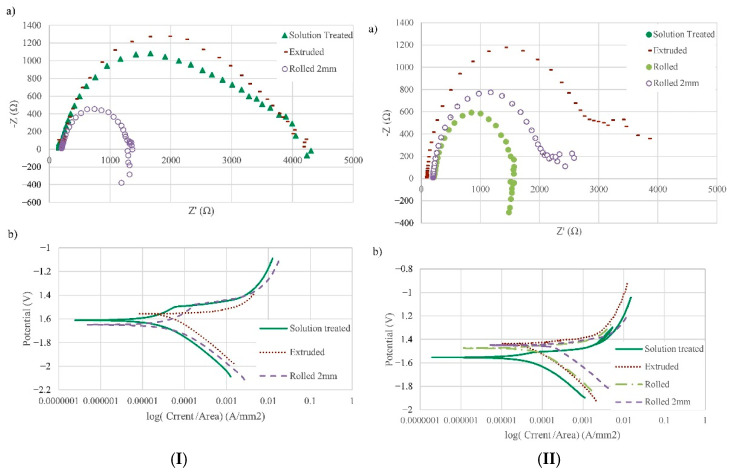
Electrochemical tests of various samples: (**I**) Mg-0.24Sn-0.04Mn alloy (**a**) EIS Nyquist plots, (**b**) potentio-dynamic curves; (**II**) Mg-0.24Sn-1.16Zn-0.04Mn alloy (**a**) EIS Nyquist plots and (**b**) potentio-dynamic curves [41].

The content and existing form of Zinc (Zn) in Mg alloy will affect the corrosion resistance obviously. The allowable limit of solid solubility of Zn in Mg is ~6.2 wt.%. With the decrease in temperature, the solid solubility will be decreased [38]. It is often added along with Zr, Al, RE, and other elements, through precipitation strengthening to achieve the purpose of enhancing Mg alloy strength. Shi et al. [39] illustrated that if the amount of Zn was usually added below 2%, it would have a negative effect on the corrosion behavior of Mg alloy when the Zn content was greater than 2.5%. Besides, the cathodic hydrogen evolution reaction of Mg alloy would be inhibited when its content was less than 1 wt.%. However, when the Zn content was increased to 5%, the alloy phase was formed, and the rate of hydrogen evolution was increased in the cathode area. At the same time, the addition of Zn would accelerate the anodic dissolution of Mg alloy. Zhang et al. [40] stated that the corrosion resistance of Mg alloys increased in the order of Mg − 0.5 wt.%Zn < Mg − 1.0 wt.%Zn ≈ Mg − 2.0 wt.%Zn as the Zn content was increased. Nahed et al. [41] found that the addition of Zn did an unbenefited effect on the corrosion rate of Mg-0.24Sn-0.04Mn and Mg-0.24Sn-1.16Zn-0.04Mn alloys. It positively shifted the potential of Mg alloy, which resulted in the reduction of corrosion rate (Figure 5). Meanwhile, Zn can reduce the impurities such as Fe and Ni. It can also improve the allowable limit of impurity Cu to a certain extent. The local corrosion tendency of Mg alloy is reduced; however, the effect of Zn is not obvious in Mg-Al alloy.

#### 3.2.2. Manganese

**Figure 6:**
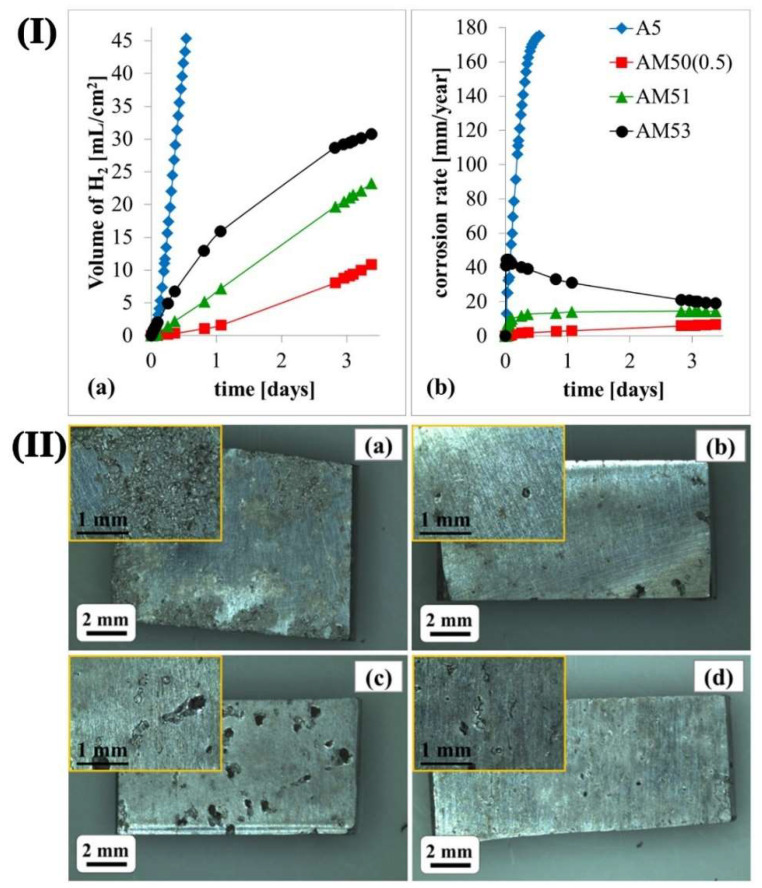
(**I**) Hydrogen evolution (**a**) and corrosion rate (**b**) for Mg-5Al-xMn alloys immersed at 3.5 wt.% NaCl solution; (**II**) Macrographs of corroded surface after 3 h immersion of Mg-5Al (**a**), Mg-5Al-0.5Mn (**b**), Mg-5Al-1.4Mn (**c**) and Mg-5Al-3.1Mn (**d**) alloys [42].

Manganese (Mn) element will enhance the corrosion resistance of Mg alloys obviously as well. It can reduce harmful impurities, refine grain size, and form a corrosion film layer induced by precipitation of various Al-Mn intermetallic with different Al/Mn ratios. However, a further increase of Mn amount results in a greater amount of the intermetallic, which increases the galvanic micro-coupling in its turn. Mn can effectively reduce the Fe impurity present in Mg alloy. They react during the melting of the Mg alloy and generate intermetallic compounds to precipitate at the bottom of the ingot. This reduces the content of Fe and the effective area of the cathode, thus improving the corrosion resistance of the Mg alloy. Polina et al. [42] proposed that the corrosion resistance was related to the ratio of Fe/Mn in the Mg alloy. Figure 6I showed that during 4d of immersion, the hydrogen evolution rate decreased significantly with the addition of 0.5 wt.% Mn compared with Mg-5Al alloy. However, the hydrogen evolution rate increased with a further increase of Mn. The corroded surfaces of Mg-5Al-xMn alloys immersion 3 h in 3.5 wt% NaCl solution were shown in Figure 6II. It is clear that the corrosion damage of Mg-5Al alloy was much more severe than the alloys with Mn. Also, Mg-5Al-1.4Mn alloy revealed deeper pits compared with other Mn-contained alloys. Baril et al. [43] showed that the corrosion behavior of AM50 was more uniform than that of AZ91. Wan et al. [44] found that not only the mechanical properties of Mg but also the corrosion resistance of the Mg-3Ni alloy were enhanced greatly after adding the Mn element. This was attributed to the solid solution of Mn, which could improve the electrode potential of the Mg alloy.

#### 3.2.3. Calcium

**Figure 7:**
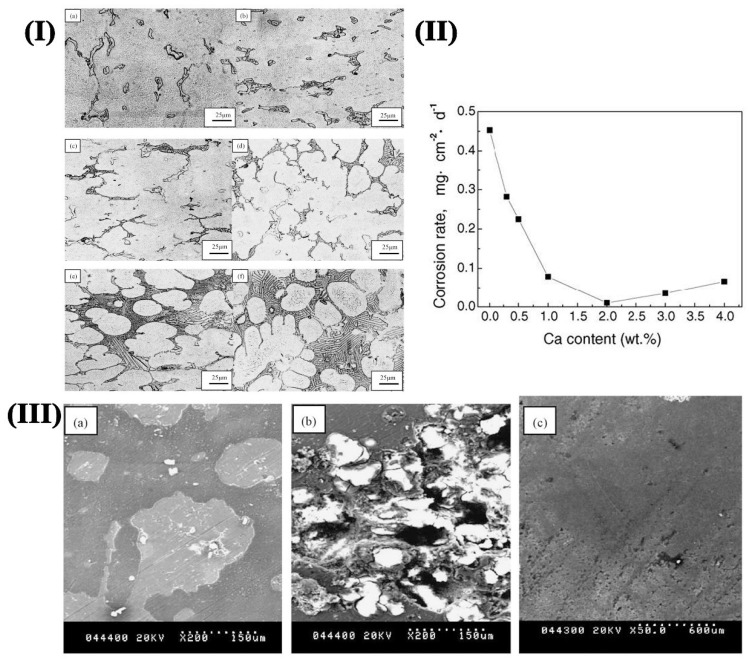
(**I**) Optical microstructure of as-cast AZC alloys (**a**) AZC03, (**b**) AZC05, (**c**) AZC1, (**d**) AZC2, (**e**) AZC3, (**f**) AZC4; (**II**) Weight loss corrosion rate for AZC; (**III**) Corrosion surface morphology: (**a**) AZ91D, (**b**) AZ91D, (**c**) AZC2 [47].

Calcium (Ca) can improve the pitting resistance and reduce the self-corrosion current density of Mg alloy. Therefore, the micro battery effect of Mg alloy is reduced, and the corrosion resistance is improved. Besides, the proper amount of Ca can refine the microstructure and improve the oxidation resistance of Mg alloy. Deng et al. [45] found that the loose MgO film on the alloy surface could be changed into a more compact MgO/CaO surface when a small amount of Ca was added to the Mg alloy, which resulted in the improvement of the corrosion resistance. However, there are different opinions about the effect of Ca on the properties of Mg alloy at room temperature. Song et al. [46] believed that the addition of 0.5% Ca reduces the corrosion rate of the AZ91 alloy at room temperature. But Wu et al. [47] found that the corrosion rate was reduced greatly when the Ca reached 1%. Besides, the strength and ductility were also improved. Figure 7I shows the microstructure of the AZ91D Mg alloy with various Ca additions. It could be identified that the size of the dendrite cell and the Mg_17_Al_12_ phase were refined obviously with the increase of Ca content. The corrosion rate (CR) of AZC alloys is shown in Figure 7II. CR of AZC alloys tends to decrease with an increase in Ca content. Thus, CR was less than 0.1 mg/cm^−2^ d^−1^, which was about 20% of AZ91D alloy. However, CR increased slowly when the amount was larger than 2%. The SEM images of the AZ91D and AZC alloys after 3d of immersion in 5 wt.% NaCl solution is shown in Figure 7III. The corrosion film ran away from the corroded surface, and the pitting corrosion was much more severe in AZ91D alloys. The best corrosion rate was obtained for AZC2 alloy mainly due to the generation of a relatively compact corrosion product layer. Further, the formation of a new Al_2_Ca phase with network distribution could increase the self-corrosion potential of Mg alloy and reduces the corrosion current density, thus impeding the Mg corrosion. When the Ca amount reaches 2%, the surface of the alloy is covered with the intact corrosion film, which provides good corrosion protection. Wang et al. [48] believed that the corrosion resistance of Mg alloys first decreased and then increased when the Ca content was increased. The standard electrode potential of Ca was −2.76 V, which was lower than that of Mg. When a small amount of Ca was added to the Mg-Mn alloy, the corrosion potential of the alloy decreased rapidly, and the corrosion resistance also decreased accordingly. When the content of Ca continued to increase, the corrosion potential of the alloy basically remained unchanged, and corrosion resistance was greatly improved owing to the appearance of the Mg_2_Ca phase. When the Ca content reached 6%, the corrosion potential of the alloy decreases, and the corrosion rate decreases to 11.8% [49]. However, the superfluous calcium compounds are harmful, especially used as stents during implanting in the blood vessel. Thus, the amount of addition should be considered carefully. 

#### 3.2.4. Rare Earth (RE) Elements 

Rare earth (RE) elements can also be usually used in Mg biomaterials, and RE element additions can refine the grain size of Mg alloy, improve toughness, and reduce porosity and hot cracking tendency during casting and plastic deformation processing. Thus, the corrosion resistance of Mg alloy could be significantly modified in RE-magnesium alloys. La, Ce, Y, Nd, Pr, etc., are commonly used in Mg alloys.

RE elements are easily turned into an oxide, forming passivated film solid solution in the matrix, thus improving the corrosion resistance of the alloy. Jin et al. [50] explained that the RE elements and Al would form a new Al-RE phase when Al was contained in the alloy, which was distributed in the intergranular of the alloy. The new phase could be passivated in a wide range of pH values. The addition of rare earth elements could play a role in removing hydrogen so that the cathodic process of Mg alloy could be inhibited by negative self-corrosion potential, and the cathodic polarization potential becomes low [51]. RE-Mg alloys could enter the surface of the alloy, which reduces the hydrogen evolution from the surface film and make the surface film more stable. Most of the rare earth elements in the alloy have a large solution limit. The solid solubility will decrease sharply with the decrease in temperature. A small amount of RE elements can form more diffuse precipitate phases, which play the role of oxygen and hydrogen removal during the alloy melting [52]. 

Y refines the grain size significantly, and the Mg_24_Y phase formed by Mg and Y affects the corrosion rate directly. Zhang et al. [53] found that the Mg-Y alloy presented uniform corrosion when the amount was less than 2.5%. The pitting corrosion would occur when the content of Y was more than 2.5%. Moreover, Du et al. [54] indicated that the addition of Y could improve the stability of the corrosion film on the sample surface. Besides, Y can also be added to Mg alloys with other alloying elements. It is found that the corrosion resistance of Mg-Zn alloy can be enhanced by adding the appropriate amount of Y to the alloy. A small amount of Ce, La, Sc, and Gd are proven to be beneficial in improving the corrosion resistance of Mg alloy. Liu et al. [55] indicated that the addition of Ce and La in AM60 could reduce the β phase and generate the RE phase with the correct potential, thus inhibiting the micro-galvanic corrosion. Yao et al. [56] pointed out that the corrosion resistance of AZ91 alloy was best when the Sc content was 0.39. The formation of the new phase Al_3_Sc consumed Al, which reduced the β phase (cathode phase) and hindered the cathodic reaction. Moreover, Sc was solidly soluble in the alloy, which enhanced the passivation ability of Mg alloys. Wang et al. [57] stated that the corrosion resistance of the ZK60 alloy was the lowest when the Gd addition content was 1.6%. MgZn and Mg_5_Gd in the second phase increased obviously and distributed in a network, which accelerated the galvanic corrosion of the alloy when the Gd was 2%.

**Figure 8:**
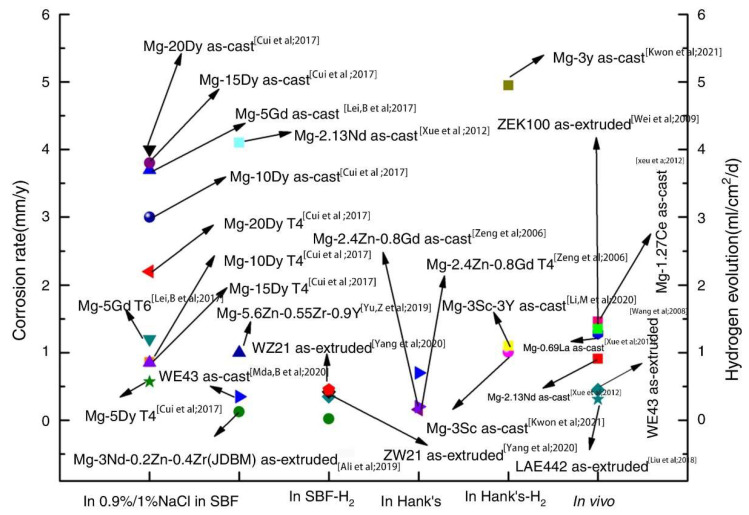
Corrosion rates of selected Mg-RE alloys in 0.9%/1% NaCl, in SBF, in Hank’s [58].

Figure 8 shows the corrosion rate and hydrogen evolution of Mg-RE alloys in 0.9%/1% NaCl during in vivo and in vitro tests [58]. The extruded LAE442 and WE43 alloys exhibited the lowest in vivo corrosion rates. Simultaneously, its strength was reduced by 40% when the decreased volume of LAE442 reached 15.2% during the three months in vivo experiment. 

However, RE elements are not necessary for the human body, which should be cautious during addition, and the toxicity test should be carried out and analyzed before implanting. 

### 3.3. Grain Refinement 

The grain size, content, and second phases will affect the corrosion resistance of Mg alloy, obviously. The higher the grain boundary energy and the more favorable nucleation sites, the more rapid formation of protective film. Therefore, the finer the grain is, the higher the grain boundary density, and the better the corrosion resistance is obtained [59]. The self-corrosion potential of the second phase is usually larger than that of the α-Mg in Mg alloy, which will accelerate the corrosion of the alloy. Partial recrystallization or complete recrystallization usually occurs in the hot working process of Mg alloys, which can refine the grain size and make the uniform and fine second phase disperse in the alloy, thus reducing the tendency to form a local corrosive battery [60]. Deformation processing and rapid solidification are the main methods to refine the grains of Mg alloy.

#### 3.3.1. Deformation Processing

Through proper deformation processing, such as rolling, extrusion, high-pressure torsion, equal channel angular pressing, etc., the microstructures are effectively refined. As a result, the open circuit corrosion potential of Mg alloy increases, and the corrosion resistance also improve to a certain extent. The common deformation methods are hot plastic deformation and superplastic deformation.

**Figure 9:**
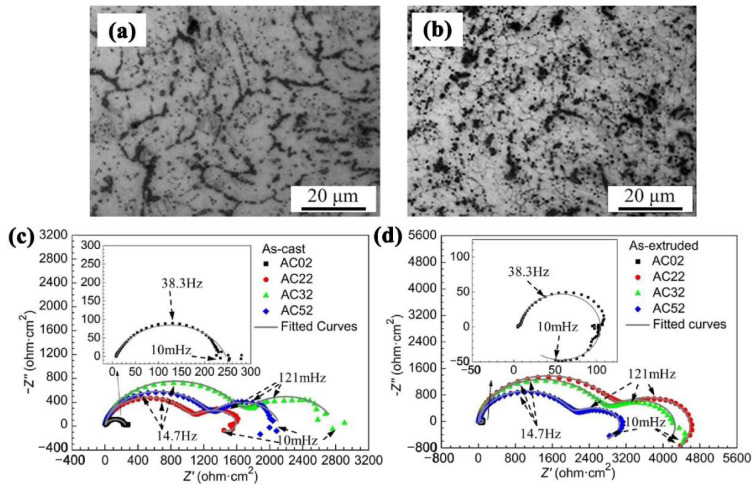
Optical micrographs of (**a**) as-cast and (**b**) as-extruded alloys; Nyquist plots of EIS (**c**) as-cast and (**d**) as-extruded alloys [62].

Hot working plastic deformation refers to the change of microstructure of Mg alloy by extrusion and rolling [61]. For example, Mg alloys with different microstructures and properties can be obtained by using different extrusion temperatures, changing extrusion ratio, and extrusion rate to improve the grain size of extruded bars. In the process of hot extrusion, the grains are elongated so that they break into tiny particles when the extrusion ratio is large. The second phase of dispersion hinders the growth of grains and results in fine grains. Wu et al. [62] investigated the effect of extrusion deformation on the corrosion behaviors of Mg-Ga-Al alloys. Figure 9a,b shows the optical microstructures of as-cast and as-extruded alloys, respectively. Compared with the as-cast alloys, grains of as-extruded alloys were obviously refined. The Nyquist plots obtained from EIS were shown in Figure 9c,d. It indicated that better corrosion resistance was obtained in as-extruded alloys. 

The conventional hot extrusion process can refine the grain size to a certain extent and reduce the corrosion resistance of Mg alloy in a simulated body fluid solution. However, this method has a small strain, limited grain refining ability, incomplete crushing, and homogenization degree [63]. The microstructure is not uniform, and the segregated phase often forms a banded structure after processing. At the same time, the recrystallized grains are easy to grow up during the processing of the alloy, so it is difficult to obtain ultrafine grains. The uneven microstructure and the second phase distribution will decrease the CR of the alloy.

Severe plastic deformation (SPD) provides the possibility for manufacturing ultrafine biological Mg alloys. During this, the accumulated true strain of Mg alloy is very large through one or more repeated plastic deformations. A large number of high-density dislocations are produced in the metal and alloy to achieve a significant grain refinement [64]. Moreover, the second phases of the alloy are refined effectively and tend to be distributed evenly to improve the comprehensive properties of Mg alloy. At present, SPD techniques used in the biological Mg alloys are mainly as follows:

(1) Equal Channel Angular Pressing (ECAP)

**Figure 10:**
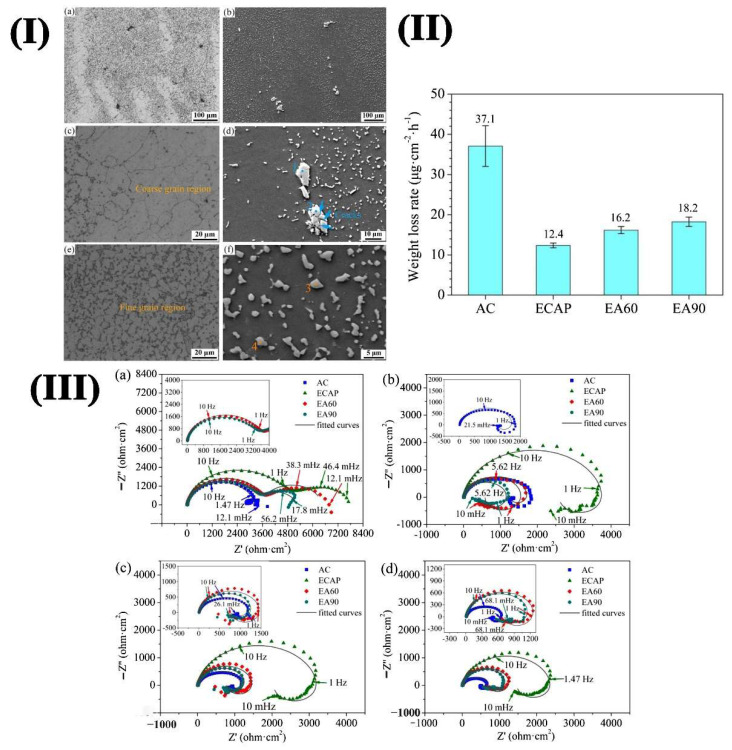
(**I**) Optical and SEM micrographs (**a**–**f**) of ECAPed AZ91 Mg alloy; (**II**) Weight loss rates after immersion in 3.5 wt.% NaCl solution for 7 d; (**III**) Nyquist spectra for various times (**a**) 1 h, (**b**) 1 d, (**c**) 3 d and (**d**) 7 d [66].

During ECAP, the processed material will be extruded through the channel where two axes intersect, and the interface size is equal [65]. In the process, a large shear strain is induced, resulting in the rearrangement of dislocations so that the grain is greatly refined. After several passes of extrusion, the microstructure of Mg alloy is uniform, and grain refinement and corrosion resistance are improved. The preparation of ultrafine Mg alloys by ECAP is relatively mature. Yang et al. [66] found that ECAP + aged AZ91 alloy with a heterogeneous structure exhibited the best corrosion resistance, which was mainly due to the reduction of β-phase particles and the weakened electrolysis effect of modification distribution. Figure 10I shows the microstructures of the ECAP alloy. As could be seen, the microstructure was refined obviously after ECAP processing. The weight loss after immersion for 7d was exhibited in Figure 10II. It was clear that the weight loss was the least for the ECAPed alloy among the four alloys. Further, the weight loss results are consistent with the EIS results, where the ECAPed sample exhibited a larger capacitive arc than as-received Mg alloys. 

However, due to the low symmetry and the low sliding system at room temperature, it is difficult for ECAP to obtain ultrafine microstructure in tightly arranged hexagonal Mg and its alloys. In addition, it is easy to micro-crack in low-temperature extrusion, which is not beneficial for improving the corrosion resistance of Mg alloy.

(2) High-Pressure Torsion (HPT)

**Figure 11:**
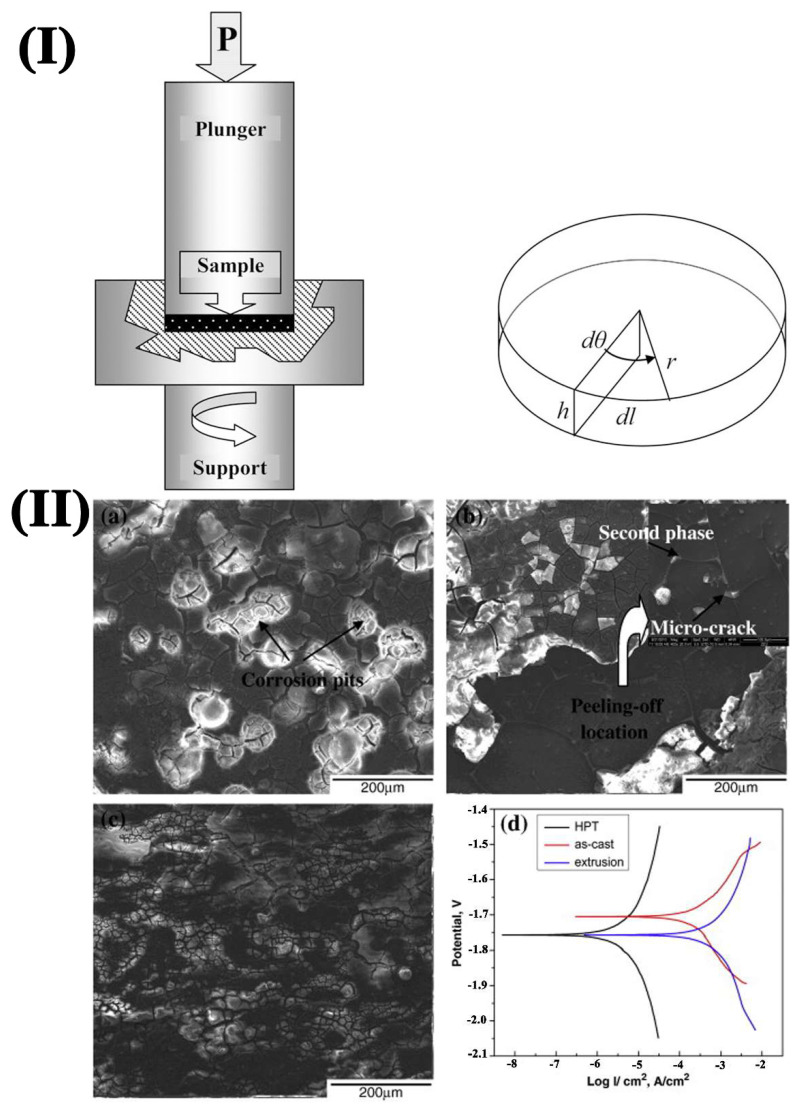
(**I**) Schematic illustration of HPT processing; [67] (**II**) SEM images of (**a**) as-cast, (**b**) convention extrusion and (**c**) HPT-treated samples after immersion for 2 days; (**d**) potentiodynamic curves [68].

High-Pressure Torsion (HPT) technology is a typical server strain plastic deformation process. During this process, a large shear strain can be introduced to the outside of the sample, causing serious torsional deformation. The schematic diagram of HPT processing is depicted in Figure 11a [67]. The sample is located between two anvils in the form of a disk where a compressive pressure and simultaneously torsional strain is subjected. Under the common influence of grain crushing, dynamic recrystallization, grain and second phase redistribution, and grain refinement are introduced to obtain uniform ultrafine grains. Dynamic recrystallization is more likely to occur due to a large amount of plastic deformation at the same time. As a result, the microstructure becomes uniform, the stress is released, and the properties of the material are improved, resulting in the alloy exhibiting ultra-high strength, excellent superplasticity, and uniform corrosion. Gao et al. [68] found that the particles trended to be nano-sized and distributed uniformly in grain interiors after HPT. The samples exhibited homogeneous corrosion during the immersion test. As shown in Figure 11b, the corrosion current density of HPT-treated alloy decreased. However, the corrosion potential shifted to a more negative value than the as-cast alloy, and the best corrosion resistance surface was obtained. However, due to the limitation of equipment conditions, HPT can only process a small slice of samples, which limits its application in biodegradable materials.

(3) Cyclic Extrusion and Pression (CEC)

**Figure 12:**
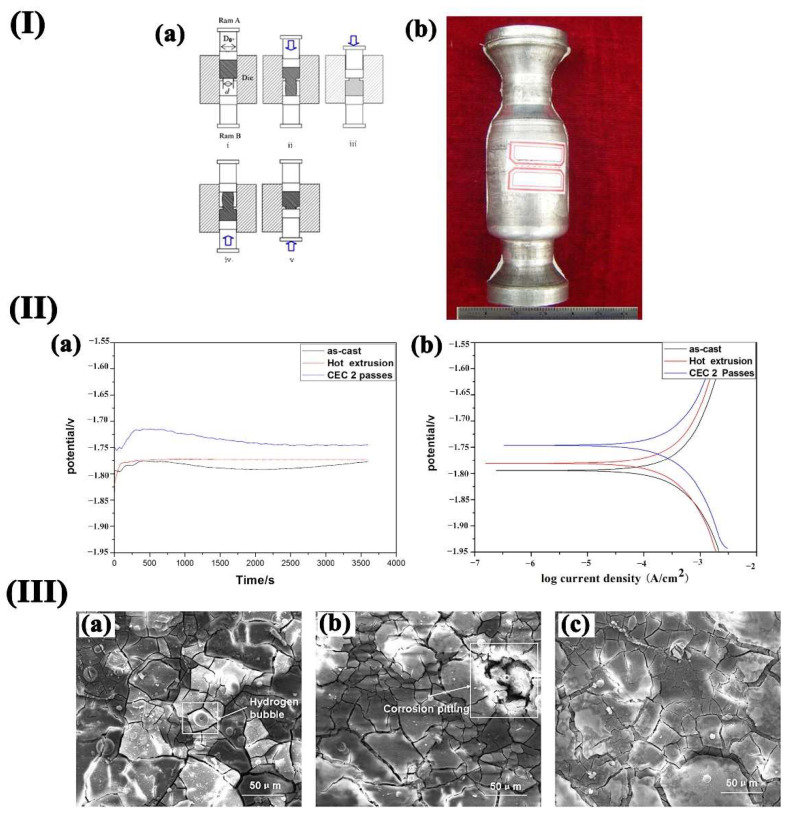
(**I**) **(a)** Schematic illustration of the CEC procedure; (**b**) 2 passes CEC specimen; (**II**) OCP (**a**) and Tafel (**b**)curves of various samples in SBF solution at 37 °C: (III) SEM images of samples after immersion for 48 h: (**a**) as-cast, (**b**) hot extruded, (**c**) CEC 2 passes [69].

The principle of the CEC process is shown in Figure 12(Ia). The 2 CEC passes sample is shown in Figure 12(Ib). The material is rubbed repeatedly by different passes of back-and-forth extrusion and compression. Under the influence of the crushing, dynamic recrystallization, and the redistribution of grains, the whole structure tends to be uniform, and grain refinement is significant. Therefore, the use of CEC to refine the grain size of structural materials can obtain uniform ultra-fine grain structure, which improves the strength, toughness, and uniform corrosion of the material. Wu et al. [69] conducted CEC to enhance the corrosion resistance of Mg-Zn-Y-Nd alloy. Figure 12(IIa) shows the potential corrosion curves (a) and the polarization curves (b) of specimen immersion in SBF. The corrosion resistance of CEC-treated alloy tends to be a more positive value than as-cast and extruded ones. Figure 12III shows the surface of the Mg–Zn–Y–Nd alloy after immersion in SBF for 48 h. There were fewer corrosion cracks than those of the as-cast and extruded alloys. This was mainly because the film layer was more compact, which resulted in better corrosion resistance. 

Using SPD technology to produce UFG structure Mg alloys is proven to be much more effective in enhancing the corrosion properties. However, the size of the samples is usually limited. Besides, most of the processes are not continuous, during which many passes are needed to refine the grains to the desired scale. Developing new SPD methods to achieve fine-grained Mg alloys is necessary to solve this problem. 

#### 3.3.2. Sub-Rapid Solidification

**Figure 13:**
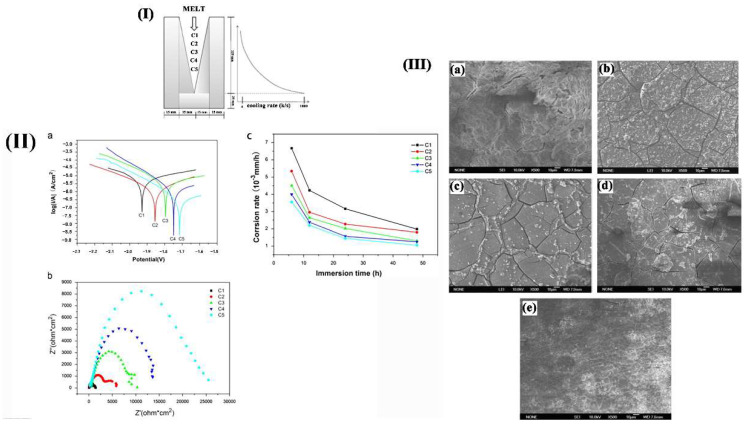
(**I**) Schematic illustration of the Cu wedge mould; (**II**) (**a**) Polarization curves and (**b**) EIS and (**c**) corrosion rate of Mg–2Zn–0.5Ca alloy with different cooling rates immersed in SBF; (**III**) Surface morphology of various cooling rates sample immersed in SBF for 72 h: (**a**) C1, (**b**) C2, (**c**) C3, (**d**) C4 and (**e**) C5 [73].

Sub-rapid solidification is a crystallization process with a cooling rate in the range of 100–103 K/s, which is between the transition zone of near equilibrium growth and far away from equilibrium growth [70,71]. It has the advantages of both rapid and conventional solidification. The solidification speed is fast, which can enhance the uniformity of microstructure and composition, reduce the segregation and defects of conventional solidification components, expand the solid solubility of alloying elements, refining the grain size and second phase. The strengthening of sub-rapid solidification is the joint strengthening of solid solution, fine grain, and dispersion effects. It will lead to harmful impurity elements solidly dissolved in the matrix instead of forming harmful precipitates. Besides, the amorphous oxide film can be formed to improve the corrosion resistance at the same time [72]. Sub-rapid solidification method can suppress local corrosion of biological Mg alloys and avoid the effect of stress caused by plastic deformation on corrosion properties. Liu et al. [73] studied the effect of solidification cooling rate on the CR of Mg–Zn–Ca alloy. Figure 13I shows the wedge-shaped copper mold used to achieve various solidification cooling rates. According to Figure 13II, the alloy exhibited higher corrosion potential when the cooling rate increased in EIS and polarization test. This suggested that the increase in solidification rate decreased susceptibility to electrochemical reaction, which was consistent with the results of corrosion rate and the corrosion surface morphology during the immersion test. Thus, it can conclude that sub-rapid solidification is an effective way to improve the corrosion properties of Mg alloys. 

### 3.4. Coating

Although the corrosion resistance of Mg alloys can be improved significantly by alloying and deformation processing, it may not meet the practical application requirements of biodegradable materials. Therefore, the study of surface modification has become a key research focus for medical Mg alloys. The biological coatings of Mg alloys mainly include bioactive ceramics and micro-arc oxidation (MAO) coatings, high polymer, chemical conversion film, metal coating, etc. [74].

(1) Bioactive ceramics

**Figure 14:**
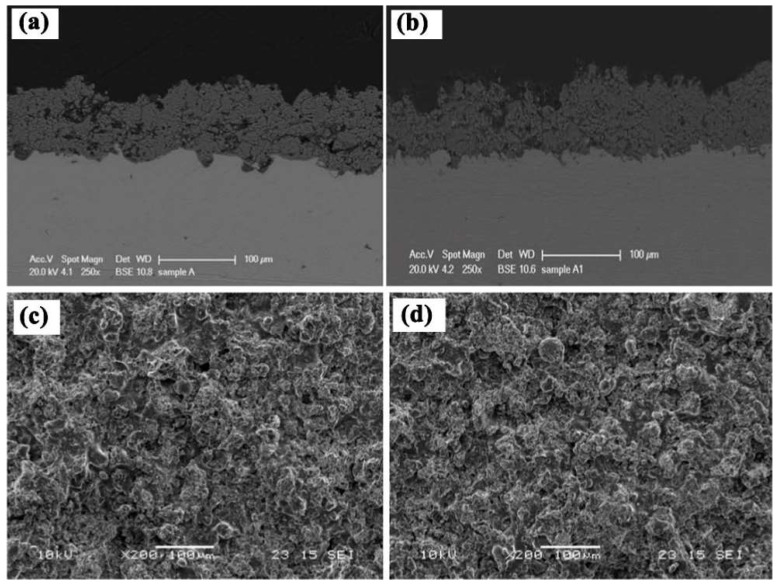
SEM micrographs of the polished cross section (**above**) and as-produced surface (**below**) of plasma sprayed *β*TCP (**a**,**c**) and HA/*β*TCP (**b**,**d**) samples [79].

Conventional bioactive ceramics mainly refers to hydroxyapatite (HA) and tricalcium phosphate (TCP) [75]. HA is the main component of human bones. It is insoluble in alkali but soluble in acid, and it hydrates weakly in an alkaline medium. Thus, HA can absorb serine-rich proteins and acidic amino acids. It is worth mentioning that HA can induce the growth of bone cells on the surface so that the cell tissue grows along the gap between the bone and the HA implant. Then the implant can be seamlessly connected with the substrate [76]. Witte et al. [77] prepared magnesium/HA composites by powder metallurgy and found that HA changed the corrosion mechanism of Mg alloy from localized to uniform type. The ceramic coating on the metal surfaces could combine the characteristics of ceramic materials and metal materials organically. It had not only the strength and toughness, machinability, electrical conductivity, and thermal conductivity of metal materials but also the advantages of high-temperature resistance, corrosion resistance, and wear resistance of ceramic materials. There were mechanical bonding and chemical bonding between the metal and ceramic coating at the same time. Owing to the chemical bonding, the metal matrix and ceramic coating bonded firmly. Harb et al. [78] studied the effect of HA and TCP additives on the anticorrosive efficiency in SBF solution. They found the beneficial role of HA and TCP-modified hybrid coating, improving both the biocompatibility and corrosion resistance. Rahul et al. [79] achieved HA/β-TCP composite coatings by plasma spray process and successive thermal treatments successfully. Figure 14 shows the SEM micrographs of the plasma spray coatings, and the average thickness of the coatings was 80–90 µm and well adherent to the Ti substrate.

(2) Micro-arc Oxidation (MAO) coating

**Figure 15:**
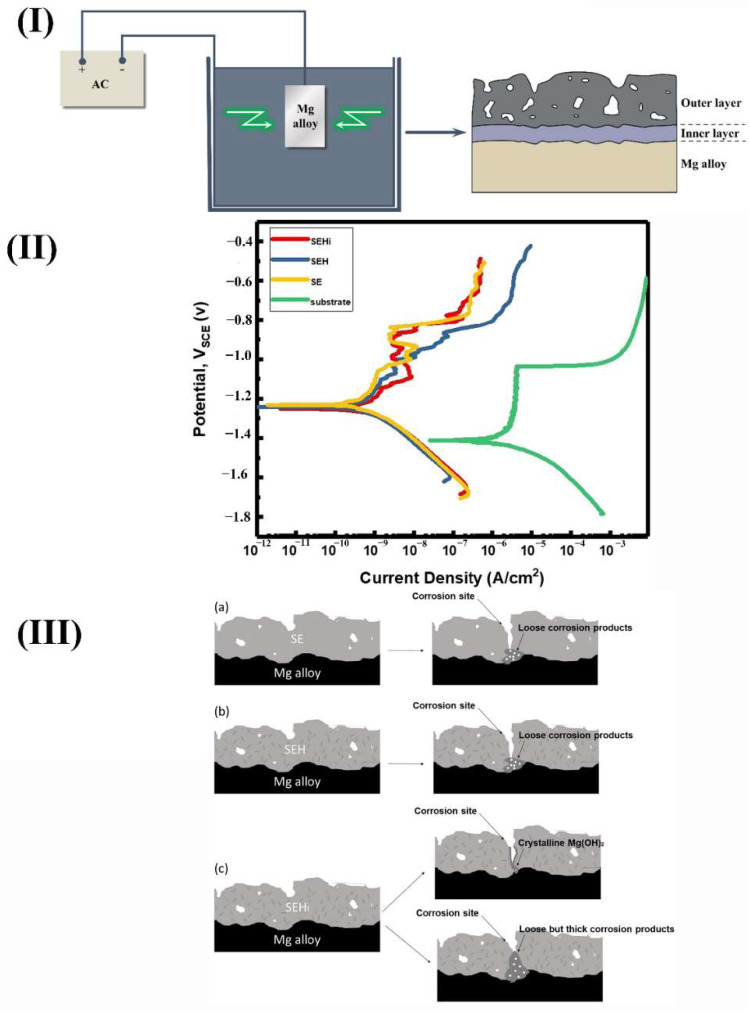
(**I**) Schematic illustration of the preparation and structure of MAO coating on Mg alloy; [82] (**II**) Potential polarization curves of the bare AZ31 Mg alloy and MAO coated samples; (**III**) the degradation and protection mechanism of three different coatings on AZ31 Mg alloy (**a**) SE (**b**) SEH (**c**) SEHi coatings [83].

The principle of MAO coating is that the material surface characteristics depend on the arc discharge to produce instantaneous high-pressure and high-temperature action. Al, Mg, Ti, and other nonferrous alloy surface were prepared with metal oxides as the main layer, doped with enhancing phase oxides as the supplementary film using specific electrical parameters in a specific electrolyte. The film has the characteristics of ceramic film, and the surface is a porous and loose structure. Figure 15I shows the preparation and structure of MAO coatings on Mg alloys. In the first step of the MAO process, a thin and dense layer was covered on Mg alloy. Then a porous layer with micro-pores and micro-cracks is deposited.

However, the corrosion resistance of the Mg alloy matrix can be improved significantly due to the existence of a dense inner layer. Jia et al. [80] prepared an oxide film with a micro-nano rough structure on Mg-1Ca alloy by MAO technology. The results showed that the corrosion current density of Mg alloy in SBF decreased by an order of magnitude, and the corrosion resistance was improved greatly. The advantages of MAO coating were excellent corrosion resistance, friction and wear resistance and good bonding ability with the matrix [81]. The rough structure of micro-nano porous was helpful for the preparation of drug-carrying coating and the growth of cell tissue. However, the corrosion mechanism of micro-arc oxidation film on Mg alloys has not been thoroughly studied, and the film forming mechanism also needs to be future deepened. Lin et al. [82] prepared a micro-arc oxidation coating with a positive protective function. Due to the self-healing effect, the enhanced corrosion resistance of 2-Abi-HNT MAO coating was obtained. Figure 15II shows the potentiodynamic polarization (PDP) curves of the bare and MAO-coated AZ31 Mg alloy after immersion. All MAO-coated samples exhibited higher E_corr_ and lowered i_corr_, indicating that the effective corrosion protection for substrate was achieved by the MAO coatings. The protection mechanisms can be seen in Figure 15III.

(3) Polymer Films

**Figure 16:**
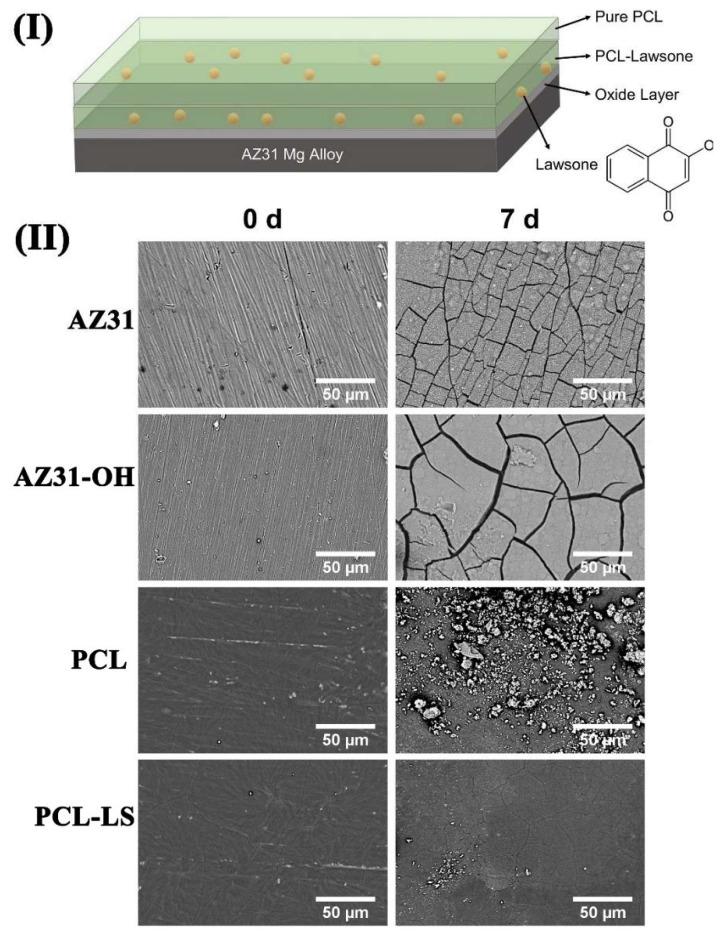
(**I**) Schematic of PCL-LS coating on AZ31 Mg substrate; (**II**) SEM images of bare, alkaline treated, and coated Mg samples before and after immersion in Hank’s solution for seven days at 37 °C [85].

Polymer bioactive materials not only have biodegradable properties, but also their corrosion resistance and physical properties can be regulated due to the regulation of their molecular formula. Polymer membranes contribute to gene expression and control cell adhesion, proliferation, and growth. Dong et al. prepared a layer of corrosion-resistant drug release film on the surface of AZ91D with composite epoxy and polycaprolactone [84]. In the beginning, a layer of epoxy with excellent corrosion resistance was prepared by drawing. Then the mixture of polycaprolactone and ibuprofen was deposited on the surface of the material through a hydrothermal reaction. When the material is implanted into the body, polycaprolactone degrades gradually, releasing ibuprofen molecules that act as an anti-inflammatory. After the polymer film was degraded completely, the epoxy resin film played a role in preventing corrosion. Asadi et al. [85] prepared an anti-corrosive coating based on polycaprolactone (PLC) and lawsone on AZ31 Mg alloys. The structure is shown in Figure 16I. The SEM observations in Figure 16II indicated that the lawsone mitigated the corrosion damages of PCL coating during immersion so that the barrier properties against the penetration of water and corrosive species were improved.

(4) Chemical Conversion Coating

The chemical conversion membrane can be divided into RE elements, organic acids, phosphates, dilute acid salts, chromate salts, KMnO_4_, etc. [86]. In conventional production, the chromate film has a good compact structure, and the chromium tissue containing structural water has a strong ability to self-repair. The amorphous histochemical transformation film of Mg alloy can be obtained in KMnO_4_ solution, and its corrosion resistance is the same as that of chromate chemical protective film [87]. Alkaline dilute acid salts can be used as early treatment for nickel plating of Mg alloy during chemical transformation reactions. In the process of a chemical conversion film treatment, the porous structure has good adsorption. What is more, the structure layer has strong adhesion, corrosion resistance, and stability in the process of nickel plating. Rudd et al. [88] prepared a protective rare earth oxide film on the surface of Mg, which improved the CR of Mg alloy significantly. However, the chemical conversion film thickness is thin and cannot resist the damage caused by friction and wear, which is generally used as a substrate. From the practical application, the chemical protective film formed by the chemical conversion method has a weak protective ability. The whole film structure is soft and thin, so it can only be used as the middle layer of the protective layer.

(5) Metallic Coating

Mg and its alloys are the most difficult metals to plate for the following reasons: Firstly, the surface of Mg alloy is easy to transform as MgO and difficult to clean, which affects the binding effect of the entire coating seriously [89]. Secondly, the electrochemical activity of Mg itself is high and is prone to be degraded in most of the plating solutions [90]. Finally, the standard potential of the whole metal coating is far beyond the matrix of the Mg alloy [91]. Any hole in the matrix has the possibility of increasing corrosion current, resulting in strong electrochemical corrosion. In view of these problems, the chemical conversion film method is used to pretreat it with metal elements, such as dipping Zn and Mn, before the metal coating. Then electroless plating and other methods increase the bonding strength of the coating by using electroplating.

**Figure 17:**
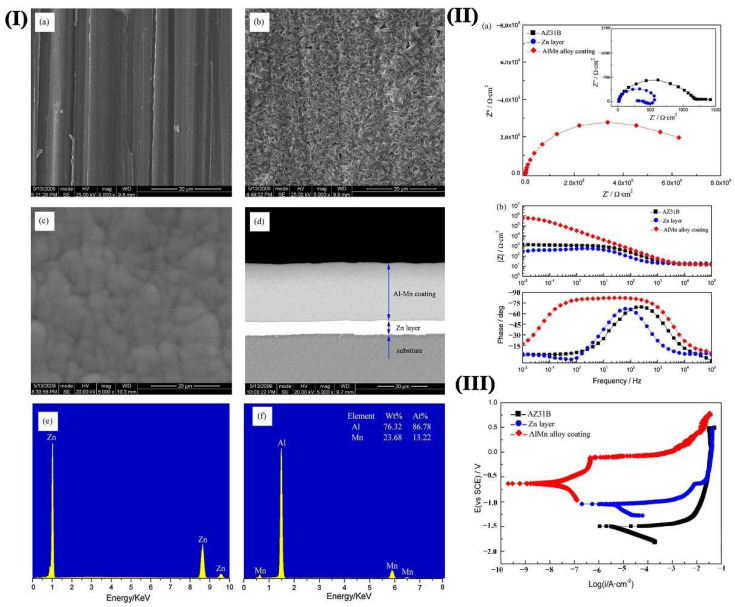
(**I**) SEM top-view micrographs of the (**a**) AZ31B substrate; (**b**) Zn layer; (**c**) Al–Mn alloy coating; (**d**) cross-section of Zn/Al–Mn alloy composite coatings; EDXS analysis of (**e**) Zn layer and (**f**) Al–Mn alloy coating; (**II**) (a) EIS plots, (b) modulus Bode plots and potentiodynamic curves (**III**) of bare AZ31B alloy, Zn layer, and Al–Mn alloy coatings in 3.5% NaCl solution [94].

Many metal coatings can be prepared on the surface of Mg alloy. Ti coating has good corrosion resistance and unique ‘biophilic’ characteristics. The coating is expected to be used as a surface-active modification layer and is widely used in medical metal materials [92]. Zn coating can improve the surface CR of Mg alloy significantly. However, it cannot meet the requirements of wear resistance. This can be used as an intermediate layer [93]. The Zn-Al composite coating has excellent performance, and subsequent treatment of the coating can significantly improve its performance. Yan et al. [94] pre-plated a Zn layer and applied an amorphous Al-Mn alloy coating on the surface of AZ31B. The results in Figure 17 showed that a high corrosion rate emerged on the single Zn layer, while it was protected by the Al–Mn alloy coating significantly. 

However, the existing coating on the surface of Mg alloy has a single function and cannot meet the requirement of various materials at the same time. Therefore, the development of multifunctional coating preparation technology with high corrosion resistance and high hardness will become the focus of future research. 

**Other factors**

(1) Stress

In some environments, such as corrosive solutions containing chromates, sulfates, and Mg alloys are highly sensitive to stress corrosion under static loads lower than their yield strength when combined with tensile stress acting on or outside the alloy. Stress corrosion usually reduces the material properties and restricts the application of Mg alloy. Alloy composition, mechanical properties, and environmental factors have serious effects on the stress corrosion cracking tendency of Mg alloys. The plastic deformation caused by stress prevents the crack tip from forming a protective film or causes the crack tip film to break continuously. The crack tip surface activity increases, thus promoting local electrochemical corrosion [95]. Stress causes the crack produced by corrosion to open the depth so that the electrolyte flows into the crack and corrosion continues. The stress increases the diffusivity of hydrogen in the metal, and the crack tip is the most stressed place, which is conducive to hydrogen entering and hydrogen embrittlement fracture.

Studies have shown that some forming processes, such as cold rolling, can improve the stress corrosion resistance of Mg alloy. However, the process of room temperature assembly and rolling will increase the residual stress inside the casting, which will make the material more sensitive. Although low-temperature annealing may increase the concentration of hydrogen in the matrix, it can effectively reduce the residual stress in the assembly process [96]. Therefore, it is still regarded as the standard treatment method to avoid stress corrosion cracking.

(2) PH value

The self-corrosion potential of Mg and its alloy gradually becomes positive with the increase of pH value, and the anode current density decreases slowly. The corrosion behavior of Mg alloy is different at pH ranges. The NaOH film formed by the Mg itself can protect the metal over a wide pH range [97]. In an acidic solution, the thermodynamic trend of the corrosion trend of H^+^ on the alloy surface decreases, and the equilibrium electrode potential of hydrogen moves in a positive direction, resulting in more uniform surface corrosion products [98]. In a neutral solution, the main factor affecting corrosion is a large amount of CO_2_ in the air, and the dissolution of CO_2_ in water can form HCO^3−^. MgCO_3_ is formed by interaction with the Mg matrix, and then uneven carbide thin film Mg_2_(OH)_2_CO_3_·3H_2_O is formed on the surface, which accelerates the corrosion of the Mg alloy [99]. In an alkaline solution, a passivation film or insoluble hydroxide is formed on the surface, which gives protection to the matrix and has certain corrosion resistance to some extent with the increase of pH value [100]. Yun et al. [101] studied the effect of pH value on the stress corrosion behavior of casting AZ91D alloy. It was proved that the fracture time of the two kinds of materials increased, and the metallic specimen was more prone to fracture with the increase of pH value under the same conditions. The reason for the difference in fracture time was the role of H^+^. With the increase of pH value, the oxide film thickness increased.

(3) Temperature

The temperature changes the microstructure of Mg alloy, thus changing its corrosion properties. With the increase in ambient temperature, the corrosion potential of AZ series Mg alloy is negative, and the corrosion rate increases [102]. Medhashree et al. [103] investigated the effects of sulfate ion concentration, temperature, and medium pH on the corrosion of Mg-Al-Zn-Mn alloy in a 30% aqueous ethylene glycol solution. The results exhibited that the rate of corrosion increased with the increased temperature of the medium.

(4) Solution Composition

Various ions present in the corrosive solution have different effects on the corrosion properties of Mg alloys. Cl^−^, SO_4_^2−^, NO^3−^, and Br^−^ can be adsorbed in the surface film to change the protection of the surface layer and accelerate the corrosion of the Mg alloy [104]. The influence of Cl^−^ on Mg corrosion has been the most studied case. Cl^−^ ions have the properties of fast motion and can penetrate the protective film of Mg. When Cl^−^ is in an aqueous solution, pitting occurs on the surface of Mg alloy, and the corrosion rate increases with the increase of the Cl^−^ concentration [105]. In the medium containing Cl^−^, the corrosion of Mg alloy shows a negative differential effect. The negative difference effect refers to the phenomenon that the self-corrosion current density of Mg alloy increases rather than decreases with the increase of anode polarization current or potential under the condition of external anode current and potential.

When the solution contains Cu, Fe, Ni, and other heavy metal salts, these heavy metal salts is reduced on the surface, precipitate out, and accelerate the corrosion rate of the Mg alloys [106]. Mg is relatively stable in either ammonium or alkali solutions, in which the ammonium salt is more corrosive to the Mg alloy. The highly oxidizing solute in the solution may react with the Mg alloy to form the low-valent compound. The formation of Mg(OH)_2_ or Mg^2+^ is related to the reduction reactions of these oxidants. Among all the solutions, the corrosion of Mg alloy in acid solution is the most severe. The degree of corrosion slows down with the decrease of acidity. Regardless of the acidity or basicity of the solution, the causticity of ions or salts to Mg varies. According to their corrosive effect on Mg, salt compounds can be divided into four groups, as shown in Table 1.

## 4. In Vivo Implantation of Mg Alloys Vascular Stent

**Figure 18:**
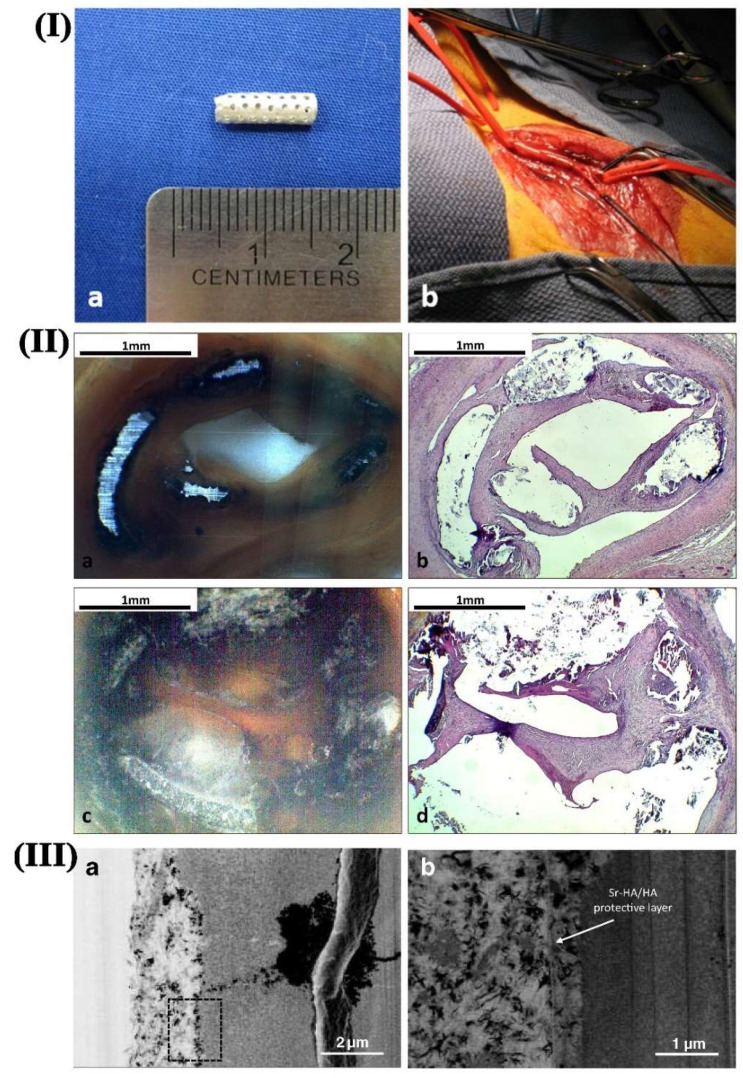
(**I**) tubular Mg-0.3Sr-0.3Ca stent (**a**) before and (**b**) after implantation into the femoral artery of the experimental animal; (**II**) Optical and histology images of vascular tissue surrounding (**a**,**b**) Mg-0.3Sr-0.3Ca and (**c**,**d**) WE43 tubular stent samples implanted in femoral artery for five weeks; (**III**) High-resolution SEM images from the interface of retrieved Mg sample after five weeks of implantation and the Sr-substituted HA layer. Image (**b**) is the magnified view of the rectangular area marked in (**a**). [111].

At present, most of the studies on biodegradable Mg alloys focus on the preparation of materials, surface modification, degradation in simulated body fluids, and other aspects. At the same time, there are few types of research on the direct implantation of such materials in vivo. Zhang et al. [109] implanted different forms of AZ31B Mg alloy into rabbits and took Ti alloys as the control group. The effects of Mg alloy on the experimental animals and their degradation behavior in vivo were researched. The results showed that bone callus was formed around the implant after two weeks of implantation of Mg alloy, and mature bone tissue was formed eight weeks later. The degradation products in vivo are mainly calcium magnesium apatite, which could be discharged from the body through metabolism. The experimental results show that the Mg alloy implantation in animals was safe at the early stage and can induce new bone formation in the matrix. Witte et al. [110] implanted Mg alloy into the femur of guinea pigs and found that the main components of degradation products were Ca, Mg, P, etc., by analyzing the surface composition of the Mg alloy. Besides, it could be found that high contents of Ca and P also appeared in addition to the inherent elements of Mg alloy. Both of these elements are necessary for the formation of new bones in the body. Their presence will further induce osteoblast differentiation and calcium salt deposition to stimulate new bone formation. Bornapour et al. [111] implanted a tubular Mg-0.3Sr-0.3Ca and WE43 stent into the femoral artery of a dog for the in vivo test. Figure 18I shows the tubular Mg-0.3Sr-0.3Ca stent (a) before and (b) after implantation. Figure 18II exhibited the optical and histological images of the cross sections of the tubular stent. It could be seen that a thin layer of endothelial cells covered the internal wall of the Mg-0.3Sr-0.3Ca stent, which prevented thrombosis and blockage of the artery. Figure 18(IIIa,IIIb) presents the high-resolution images of the implanted sample as well as the protective layer formed on the surface. It was found that a thin flaky layer of calcium phosphate apatite was covered on the Mg implant. Besides, a significant amount of MgO and Sr was contained on the outermost layer. This indicates that Mg medical devices are suited to be implanted into the animal body, which are safe and biocompatible. However, there are also some problems that should be overcome thoroughly in vivo. The summary of in vivo testing carried out in animal models is highlighted in Table 2.

## 5. Future Research Trends and Challenges

Generally speaking, the nature of the material itself affects its function and service life. However, the surface properties of materials, such as chemical composition, surface morphology, and surface roughness, will affect the biological effect between materials and the organism. In addition, the effects of degradation products should be considered for degradable materials. At present, there are few studies on the compatibility of Mg alloy endothelial cells, and there are still many aspects that need to be further studied. For example, the surface morphology, surface roughness, surface charge, and surface energy of Mg alloy affect the growth and migration of endothelial cells. These results can better guide the researchers to adjust and optimize the direction of Mg alloys.

## 6. Summary

In this paper, the characteristics and development status of biomedical Mg alloys were reviewed. The corrosion mechanism and types of Mg and its alloys were summarized. Galvanic corrosion, intergranular corrosion, pitting corrosion, etc., are common in Mg alloys. However, the corrosion mechanisms are still not well understood, especially during its service, which should be further investigated. Purification can reduce the presence of impurity elements in the alloy, reducing the possibility of micro-galvanic corrosion and improving the corrosion resistance of the matrix. Though many purification methods for Mg and Mg alloys have been applied, there is no method that can remove all the impurities, and this needs to be further investigated deeply. The addition of Zn, Mn, Ca, Re, and other alloying elements in the Mg alloy can effectively hinder the precipitation of grain boundary in the second phase and improve the electrode potential of the matrix. Meanwhile, the concentration of alloying elements on the surface increases the density of the oxide film on the surface, which has a better protective effect on the matrix. However, the concentration of micro-alloying elements should be carefully controlled. Superfluous compounds are harmful, especially used as stents during implanting in the blood vessel. The critical value of various elements should be defined further. Extrusion, rolling, SPD, and other deformation processes can effectively refine the grain of the alloy. Partial or complete recrystallization occurs in the hot working process of the alloy. The fine and uniform second phase is dispersed in the matrix, which reduces the tendency of local corrosion. Nevertheless, most SPD technology can only produce small-size samples. Besides, the processes are not continuous. Developing new SPD methods to achieve fine-grained Mg alloys is necessary to solve this problem. 

Surface modification technology is the focus of biomedical Mg alloy research. At present, the coatings used for medical Mg alloys mainly include bioactive ceramics, MAO coatings, high polymer, chemical conversion film, and metal coating. At the same time, the degradation rate of Mg alloy is not only related to its purity and grain size but also affected by other factors such as stress, pH value, solution temperature, and composition of the solution. However, the existing coating on the surface of Mg alloy has a single function and cannot meet the requirement of various materials at the same time. Therefore, the development of multifunctional coating preparation technology with high corrosion resistance and high hardness will become the focus of future research.

In vivo implantation and cytocompatibility experiments on Mg alloys can effectively evaluate new biomaterials. It can be used for effective screening and prediction in biomaterial research. At present, the research on biodegradable Mg alloy materials mainly focuses on the material itself. The study of biodegradable Mg alloys in the medical field should focus on the improvement of their comprehensive properties and service conditions. This kind of material still has great potential to be developed.

## Figures and Tables

**Table 1:** The classification of salt compounds according to the corrosion effect [107,108].

Classify	Salt Compounds
Strong passivator	Fluoride, Chromate
Medium passivator	Alkali, Carbonate, Borate, Phosphate
Medium etchant	Sulfate, Acetate, Nitrate
Pitting corrosion accelerant	Chloride, Bromide, Perchlorate

**Table 2:** Biodegradation and new bone formation of Mg implants in animal models.

Alloy	Animal Model	Number of Days Implanted	Findings	Reference
ZEK100	Rabbit tibiae	9 months	Pathological effects on host tissue	[112]
Mg-Y-Nd-HRE	Rats Femora	6 months	Bone-implant interface strength Mg > Ti	[113]
Mg-6Zn	Rabbit Femora	14 weeks	87% degradation rate	[114]
Mg-0.8Ca	Rabbit tibia screw	8 weeks	Mg-0.8Ca = SS336L at 8th week Inflammation in Mg-0.8Ca > SS336L	[115]
Mg-Zn-Ca	Mice Renal vessel occlusion	4 weeks	Renal vessel was completely closed without any adverse effects	[116]
Mg PEO coating Ti	Mini pig Rivet screw	24 weeks	Bone density and BIC Ti > coated > uncoated Mg	[117]

## Data Availability

Not applicable.
